# Research designs and instruments to detect physiotherapy overuse of low-value care services in low back pain management: a scoping review protocol

**DOI:** 10.1186/s13643-022-02083-3

**Published:** 2022-10-05

**Authors:** Lukas Kühn, Lara Lindert, Kyung-Eun Choi

**Affiliations:** Brandenburg Medical School Theodor Fontane, Center for Health Services Research, Seebad 82/83, 15562 Rüdersdorf bei Berlin, Neuruppin, Germany

**Keywords:** Low back pain, Medical overuse, Health services overuse, Low-value care, Appropriateness of care, Physiotherapy, Physical therapy

## Abstract

**Background:**

The provision of low-value care services in low back pain management is a problem of global scope. Inappropriate imaging, overmedication, and overused invasive therapies are prevalent in physician services. Yet, little is known about overused low-value physiotherapy services. Most studies addressing physiotherapy overuse in low back pain management arose from countries in which physiotherapy is established in primary care. However, measures and instruments addressing physiotherapy overuse limitedly fit legislative conditions of health systems in which physiotherapy is a service of secondary care. Thus, this scoping review’s purpose is to map existing research designs and instruments aiming to detect overused low-value physiotherapy services taking specific healthcare settings and aspects of medical overuse into account.

**Methods:**

The development of this scoping review is guided by the Arksey and O’Malley framework. A two-step, peer-reviewed search strategy in accordance with the PRESS checklist will be conducted on MEDLINE (PubMed), Web of Science, and CINHAL. Additionally, gray literature will be searched on Google Scholar. Preprints of empirical studies will be included. Initially, two reviewers will independently screen articles for eligibility by title and abstract. A third reviewer will mediate discrepancies. Uncertainties will be eliminated by a full-text analysis or by contacting the corresponding authors. A four-step analytical process will guide result reporting focusing on major research questions outlined in this protocol. Numerical and narrative tables, graphics, and narrative summaries will be the methods to summarize and collate results. In the final step, the German health system will serve as an exemplary setting and frame to practically apply results.

**Discussion:**

Results of this scoping review will help researchers to systematically select overuse measures referring to aspects of the overuse typology, specific healthcare settings, and physiotherapy services. It will further provide information on the limitations of present studies and will give advice on how to address them. Moreover, this review will illustrate to what degree existing studies succeed to comprehensively cover the concept of the overuse typology.

**Systematic review registration:**

This protocol has been registered on the open science framework (https://doi.org/10.17605/OSF.IO/PMF2G).

**Supplementary Information:**

The online version contains supplementary material available at 10.1186/s13643-022-02083-3.

## Background

Globally, low back pain (LBP) represents the main cause of disability [1]. In LBP management, physiotherapy (PT) holds a key role as it is a frequently utilized health care service [2]. However, existing studies indicate a prevalence of overused low-value PT services. Across 19 countries, 35% of applied PT services for musculoskeletal health conditions are estimated to follow no evidence-based guideline recommendation [3]. In LBP, 28% of delivered services were considered to be inappropriate [4]. Medical overuse is defined as care of poor quality, which benefits do not outweigh its harms, which is less cost-effective than alternative treatments, and which does not meet patient preferences [5, 6]. At the current stage of research, a common definition of medical overuse is still lacking and is also described in other terms such as low-value care trying to address the same complex phenomenon [6]. Unfortunately, most studies addressing PT overuse concern countries with direct access legislation [7]. In Germany, direct access to PT services is still a goal to achieve and thus limits the options of PT overuse measures since authorities of independent diagnostics and medication prescribing are not part of occupational curricula and PTs act on physicians’ prescribing authorities which limits methodical approaches to investigate medical overuse in respective healthcare settings [8]. Due to unique legal regulations for PT services in Germany, this scoping review aims to map existing research approaches addressing PT overuse in LBP management fitting primary and secondary care conditions.

Verkerk et al. [6] provided a typology of low-value care services to give guidance on de-implementation in settings of prevalent medical overuse conditions. This typology comprises domains of healthcare delivery which is ineffective, inefficient, and misaligned with patient preferences and which can be approached from several perspectives: From a clinician perspective, ineffective care lacks evidence-based benefits, has the potential to cause harm, and its benefits do not outweigh its risk. From a societal perspective, inefficient services are effective in nature but modes of delivery, frequencies, and intensities of services produce no measurable patient benefits and have the potential to produce preventable costs. From a patient perspective, misaligned services are effective in nature but do not match patient preferences [6, 9].

Unfortunately, most studies investigating medical overuse of PT services for LBP focus on aspects of ineffective care [3, 10]. In this regard, authors use terms such as *appropriateness of care*, *overtreatment*, *overdiagnosis*, *overmedication*, *guideline adherence*, or *medical misuse* to approximate the complexity of medical overuse of low-value care services.

Data sources and measures to detect aspects of medical overuse are described in direct and indirect measures: Direct measures contain audits of clinical registries or patient records. Indirect measures contain quality indicator applications, survey studies of patients and healthcare providers, or claims data analysis of regional differences in healthcare delivery [9]. However, research on medical overuse detection of low-value care is still in its early stages: The authors of a critical review identified that most of the overuse measures for LBP management aimed at imaging services [11], but even there, an international consent of definitions and measures of imaging appropriateness in LBP is still not achieved [12]. Despite detections of inappropriate imaging services, a lacking availability of systematically collected administrative patient-level data still prohibits comprehensive overuse measures and fails to provide information about motives for individual low-value service provision [13]. In PT services, the identification of suitable overuse measures is even more complex since measures predominantly need to focus on therapeutic courses. Especially in countries with limited PT access, diagnostic quality indicators fail to provide needed information about PT overuse in LBP management.

Thus, this scoping review aims to contribute to the field of medical overuse research by systematically mapping existing designs and instruments detecting domains of PT overuse of low-value services in LBP management in healthcare conditions with and without direct PT access.

## Methods

### Design and registration

This study will be conducted by a multidisciplinary team with proven expertise in clinical PT, health services research, and rehabilitation sciences. The study design is following the Arksey and O’Malley framework [14] and comprises five consecutive steps: (I) identification of the research question, (II) identification of relevant studies, (III) study selection, (IV) data charting, and (V) compiling and reporting of results. Study execution will be reported in concordance with the established reporting guidelines for Scoping Reviews (PRISMA P; PRISMA ScR) (see Additional file 1 for reporting checklist of the study protocol PRISMA P) [15, 16]. In terms of research transparency, this protocol has been registered on the Open Science Framework database.

### Stage 1: Identifying the research question

The rationale of this scoping review is directed by the following research questions:

- RQ1: How is medical overuse of PT services in LBP management being measured?
- RQ2: To what extent are domains of the low-value care typology introduced by Verkerk et al. equally approached and represented?
- RQ3: Which methods fit the legal conditions of the German healthcare system?

### Stage 2: Identifying relevant studies (eligibility criteria)

The eligibility criteria of this scoping review will follow the Population-Concept-Context (PCC) framework recommended by the Joanna Briggs Institute [17]. The PCC framework of this protocol is shown in Table 1 and is extended by the additional domain of “types of evidence.”

**Table 1:** PCC framework

Criteria	Characteristics
Population	-LBP patients (ICD: M54.3; M54.4; M54.5; M54.8; M54.9) -All stadiums of LBP
Concept	-All studies aiming to detect medical overuse of physiotherapy care in low back pain management regarding effectiveness, treatment efficacy, and alignment of care
Context	-Physiotherapy care across all sectors of health services (inpatient, outpatient, and rehabilitation healthcare settings)
Types of evidence	-All types of empirical studies -Studies across all countries -Articles published in English or German language -Published and unpublished studies

The scoping review will include LBP patients according to the International Classification of Diseases (ICD) Version 10 (M.54). Specifically, diagnostics of ischialgia (M54.3), lumboischialgia (M54.4), back pain (M54.5), other back pain (M54.8), and unspecified back pain (M54.9) will be included. Studies investigating medical overuse of PT care for specific back pain events containing nerve compressions, fractures, spinal cancer, traumata, infectious diseases, or musculoskeletal birth anomalies will be excluded. According to classifications of time or severity of pain, all stadiums of LBP (acute, sub-acute, chronic, and recurrent) [18] and all grades of chronic pain severity established by von Korff et al. [19] will be included.

Regarding the concept of medical overuse, all studies aiming to detect overuse of low-value PT care in LBP management will be included and subsequently categorized into domains of the continuum of care model introduced by Michaleff et al. [9]. This model represents a tailored and continued development of the overuse typology of Verkerk et al. [6] fitting the context of PT care for musculoskeletal health conditions. Given the objective to provide an overview of existing evidence of medical overuse measures, studies of all sectors of PT care (inpatient, outpatient, and rehabilitative care settings) will be included. The time period of included publications will not follow any restrictions and will reach up until October 2021. Despite this, the authors will include articles from all regions and countries in the world.

This review will include all types of experimental studies, quasi-experimental studies, analytical observational studies, cross-sectional studies, and all types of systematic or non-systematic review studies. To avoid publication bias, available preprint studies will be included. Additionally, gray literature such as health insurance reports or other reports of governmental entities will be included. Opinion papers, editorials, commentaries, and case reports will be excluded as these article types do not provide requested information on conducted research designs and instruments. A comprehensive overview of eligibility criteria for included articles is listed below.

#### Inclusion criteria

To be eligible for this review, articles must comply with the following criteria:

- Population of LBP patients (ICD-10: M54 group)
- Studies investigating any aspect of medical overuse in the context of PT care
- Studies conducted in any health care sector
- Articles published at any time until 10/2021
- Studies conducted in any country or region of the world
- Studies reported in the English or German language
- Any studies, regardless of their study design
- Health insurance reports or reports of governmental entities

#### Exclusion criteria

Articles meeting the following criteria will be excluded:

- Articles including patients with specific back pain conditions
- Articles that do not measure aspects of PT overuse

### Stage 3: Study selection (search strategy)

Databases for the selection of eligible studies will be MEDLINE (PubMed), Web of Science, CINHAL, and Google Scholar. The search strategy will contain keywords and subject headings from referring domains of the PCC framework. Within domains, keywords and index terms will be combined with the Boolean operator “OR.” If applicable, keywords and index terms will be truncated. To connect domains, the operator “AND” will be applied. The search strategy will follow a two-step study selection approach and is led by the Joanna Briggs Institute’s (JBI) Reviewer Manual [17]. In step one, an initial, limited search of set databases will be conducted with predefined keywords and index terms (see Additional file 2) which will be used to screen retrieved articles for additional keywords and index terms. In step two, a second search including all identified keywords and index terms will be performed. The study selection process will be peer-reviewed by applying the Peer Review of Electronic Search Strategies (PRESS) checklist [20]. Retrieved articles will be imported to Endnote20 (Philadelphia, USA).

The screening process of included articles will be performed in two consecutive steps: First, two reviewers (LK, LL) will independently conduct a title and abstract screening by consensus. To solve disagreements, it will be referred to a third party (AC). Primary reasons for article exclusion will be recorded. Secondly, a full-text screening will be conducted following the principles of step one. To manage the study selection process, the online application RAYYAN Version 2021 (Cambridge, USA) will be used. A comprehensive flow of the study selection process will be illustrated by the PRISMA-ScR flowchart.

### Stage 4: Data charting (data extraction)

To guarantee an appropriate illustration of study characteristics, a preliminary data extraction chart tailored to the objectives of this review is illustrated in Table 2. The chart is developed in concordance with JBI’s Reviewer Manual [17]. Chart elements are complemented by associated questions which are inspired by recently published Scoping Review Protocols [21–23]. The data extraction process will be performed in Microsoft Excel. The chart will be piloted by two researchers (LK, LL) and adjusted if necessary. As this is an iterative process, further adjustments at later points in time will also be taken into account. One researcher (LK) will take the lead in data extraction and will be checked by a second reviewer (LL).

**Table 2:** Preliminary data extraction chart

Chart elements	Associated questions
**Publication details**	
Author(s)	Who wrote the article?
Year of publication	When was the article published?
Country of origin	Where was the study conducted and published?
Type of PT access	How are the legislative conditions for PT services in the country of origin? How is the access to PT services regulated? What occupational rights do therapists have?
Publication type	What type of publication is this? (empirical study, gray literature)
**Study details**	
Aims/purpose	What were the aims of the study?
Methodological design	What methodological design was applied?
Population and sample size	Who was the target population and how many were included in the study?
PT services	Which PT services were of primary interest?
**Conceptualization and measurement details**	
Medical overuse definition	How is medical overuse of PT services in LBP management conceptualized?
Conceptual framework	Is the concept based on a practical/theoretical framework, theory, or typology?
Domains and perspectives of the low-value care typology	Which domains of the low-value care typology introduced by Verkerk et al. [6] are investigated? (domains: ineffective care, inefficient care, misaligned care; perspectives: provider, society, patient)
Number of domains and perspectives	How many domains and perspectives of the low-value care typology were included?
Measurement	What research instrument was used for measurement (e.g., quality indicators, scaling, questionnaire, interview guide)? What level of measurement was used (nominal, ordinal, interval, ratio)? Were measures of direct or indirect nature?
Instrument psychometric properties	If applicable: Was the instrument validated? What was the reliability statistic? What was the content validity? Which guidelines or evidence synthesis were referenced to set thresholds
Limitations and challenges	Were there any reported limitations or quality issues (not a critical appraisal)?
Recommendations for further studies?	Were there any recommendations for further studies?

### Stage 5: Collating, summarizing, and reporting results

To guide the report of results, this stage will contain four consecutive steps. In the first step, a narrative summary comprising a numerical table about generic details of included articles will be provided. The table will be led by the domains of the PCC framework and will further contain information about the number of included articles, study designs, instruments, and measures, investigated PT services, LBP stadiums, and referred guideline recommendations.

In step 2, the first research question (*RQ1: How is medical overuse of PT services in LBP management being measured?)* will be addressed. An in-depth narrative description of identified measures and instruments will be provided. In an illustrative form (bubble chart or comparable figure), trends of methodical approaches for specific aspects of PT overuse, specific PT services, or specific healthcare settings will be visualized. Result reporting of *RQ1* will be completed by a narrative summary connecting research trends with reported limitations and recommendations (with a special focus on instrument validity for overused low-value PT services exposure) for future research intends.

In step 3, result reporting of *RQ2* (*To what extent are domains of the medical overuse typology equally approached and represented*?) will be approached by a narrative description of the numerical distribution of domains. Additionally, the development of a pie chart is intended to illustrate results. The chart also aims to highlight distributional differences in relation to specific PT services (e.g., diagnostics, treatments, prescriptions, advice) and healthcare settings (primary and secondary care). This is to identify potential trends of researched domains and perspectives of the medical overuse typology with referral to specific services and contexts. Thus, this potentially shows in which situations scientific methods measuring overuse domains are insufficient or not available.

In step 4 of the analysis, findings will dichotomously be mapped to health systems in which PT services are provided in primary or secondary care. Since this review particularly focuses on overuse measures for secondary care conditions, the German health system setting will serve as an exemplary context to pragmatically tailor and collate results. In this regard, a framework will be developed which assigns identified measures and results of this review to the occupational law of German PTs. In this way, we attempt to answer *RQ3* (*Which methods fit the legal conditions of the German healthcare system*?).

## Discussion

Medical overuse of low-value care services is a problem of global scope [24, 25]. In LBP care, overuse and drivers of overuse are predominantly reported for physician services [26–30] although PT is determined by intensive patient contacts along with rehabilitation. Studies addressing PT overuse of low-value services frequently concentrate on ineffective care as it appears to be less complex to distinguish services in binary categories of “good” and “bad” [3, 4]. However, these measures do not provide sufficient information on how care was delivered. Was it delivered efficiently and aligned to patient preferences? And if so, how do we manage destructive patient preferences? These aspects are of even more relevance for PT services provided in secondary care settings as quality indicators or other types of binary overuse measures referring to diagnostics, prescribing patterns, or treatment choices only have limited applicability. Due to the multifaceted complexity of the medical overuse typology and unestablished academic research traditions, countries like Germany fail to supply valid information on overused low-value PT services in LBP management [31]. Thus, this review aims to contribute to medical overuse research by providing guidance in the choice of research methods taking healthcare settings, PT services, and specific domains of medical overuse into account.

To the best of our knowledge, this review is the first of its kind and will contribute to a sustainable application of PT services by putting evidence-based, patient-centered care into the primacy of action.

## Supplementary Information

**Additional file 1.** PRISMA-P (Preferred Reporting Items for Systematic review and Meta-Analysis Protocols) 2015 checklist: recommended items to address in a systematic review protocol*.

**Additional file 2.** Initial search strategy.

## Data Availability

Not applicable.

## Abbreviations

ICD: International Classification of Diseases
LBP: Low back pain
PCC: Participants, Concept, Context
PT: Physiotherapy
