# Supplementary material for: Research designs and instruments to detect physiotherapy overuse of low-value care services in low back pain management: a scoping review protocol
Source: Syst Rev. 2022 Oct 5;11:212. doi: 10.1186/s13643-022-02083-3 (PMC9536019; doi:10.1186/s13643-022-02083-3)
Supplement: Supplementary file 2 — Additional file 2. Initial search strategy. [file 13643_2022_2083_MOESM2_ESM.docx]

**Supplementary file 2:** Initial search strategy

|  | **Participants** | **Concept** | **Context** | **Context** |
| --- | --- | --- | --- | --- |
| **Key concepts**  (key concepts will be connected by the Boolean operator “AND”) | **Low back pain patients** | **Medical overuse** | **Physiotherapy** | **All healthcare sectors*** |
| **Free text terms / natural language terms**  (synonyms, UK/US terminology, medical/laymen’s terms, *acronyms/abbreviations,* truncations) | low back pain - *LBP* [OR] back pain - *BP* [OR] acute low back pain - *ALBP* [OR] chronic low back pain - *CLBP* [OR] sub-acute low back pain [OR] lumbag*o*s [OR] backache*e*es [OR] unspecif*ic*ied back pain [OR] other back pain [OR] lumboischialgia  **Explode:** musculoskeletal condition*s [OR] musculoskeletal disorder*s [OR] musculoskeletal disease*s [OR] musculoskeletal system | overus*e*ed [OR] medical overuse [OR] overused care [OR] overused services [OR] overused health services [OR] low-value care [OR] overtreatment [OR] overdiagnosis [OR] inappropriate care [OR] appropriateness of care [OR] misus*e*ed [OR] misused care [OR] misused services [OR] high-value care [OR] guideline adherence | physiotherap*y*ies - *PT* [OR] physical therap*y;ies [OR] manual therap*y*ies [OR] non-invasive therap*y*ies [OR] exercise therap*y*ies [OR] medical gymnastics [OR] manipulation [OR] spinal manipulation [OR] physical medicin*e*es [OR] occupational therap*y*ies [OR] physiotherapis*t*ts [OR] occupational therapis*t*ts [OR] physio [OR] active treatment [OR] exercise treatment | outpatient [OR] inpatient [OR] primary care [OR] secondary care [OR] rehabilitation [OR] rehabilitative [OR] rehabilitative care [OR] ambulatory [OR] ambulatory care [OR] stationary [OR] stationary care |
| **Controlled vocabulary** **terms / Subject terms**  (MeSH terms, Entry terms) | low back pain [OR] low back ache [OR] postural low back pain [OR] recurrent low back pain [OR] lumbago | Medical Overuse [OR] Health Services Overuse [OR] Health Services Overutilization [OR] Medical Preference Misdiagnosis [OR] Overdiagnosis [OR] Overtreatment [OR] Overmedication [OR] Overuse [OR] Overutilization of Health Services [OR] Preference Misdiagnosis [OR] Unwanted Medical Care [OR] Inappropriate Prescribing | Physical Therapy Modalities [OR] Physical Therapy Techniques [OR] Physical Therapy [OR] Physiotherapy [OR] Physiotherapy Techniques [OR] Physical Therapist*s [OR] Physiotherapist*s | Ambulatory Care [OR] Outpatient [OR] Outpatient Care [OR] Outpatient Health Services [OR] Outpatient Services [OR] Services [OR] Outpatient Health [OR] Urgent Care [OR] Primary Care [OR] Primary Health Care [OR] Secondary Care [OR] Rehabilitation [OR] |

*the context section “all healthcare sectors” will only be applied if the number of search results extends study screening practicability
